# Self-Calibration of Nonlinear Signal Model for Angular Position Sensors by Model-Based Automatic Search Algorithm

**DOI:** 10.3390/s19122760

**Published:** 2019-06-19

**Authors:** Zhenyi Gao, Bin Zhou, Bo Hou, Chao Li, Qi Wei, Rong Zhang

**Affiliations:** 1 Engineering Research Center for Navigation Technology, Department of Precision Instrument, Tsinghua University, Beijing 100084, China; gaozy17@mails.tsinghua.edu.cn (Z.G.); houb15@mails.tsinghua.edu.cn (B.H.); cli16@mails.tsinghua.edu.cn (C.L.); 2 Department of Electronic Engineering, Tsinghua University, Beijing 100084, China

**Keywords:** model-based automatic search, nonlinear signal model, signal flow network, angular position sensor

## Abstract

This study proposes a novel model-based automatic search algorithm to realize the self-calibration of nonlinear signal model for angular position sensors. In some high-precision angular position sensors, nonlinearity of the signal model is the main source of errors and cannot be handled effectively. By constructing a signal flow network framework and by embedding a modeling search network, the parameters of the nonlinear signal model can be searched, and the calibration signal can be obtained. The convergence of the network search process was analyzed. The relationship between the optimization threshold and the convergence accuracy was also studied in simulations. Compared with the maximum angular error reduction to 47.42% after the calibration with simplified model that ignores signal nonlinearities, the proposed scheme was able to reduce this error to 0.0025% in simulations. By implementing the technique in a capacitive angular position sensor, the experimental results showed that the maximum angular error was reduced to 1.63% compared to a reduction of 86.02% achieved with the simplified model calibration. The effects of the search network order and layer number on the calibration accuracy were also analyzed, and the optimal parameters under experimental conditions were obtained. Correspondingly, the proposed scheme is able to handle calibration of nonlinear signal model and further improve sensor accuracy.

## 1. Introduction

Angular position information is important in many control and inspection systems. In application scenarios, such as in unmanned aerial vehicles and industrial robots, obtaining accurate angular information is crucial. Ideally, angular position sensors such as resolvers [1,2] and capacitive angular position sensors (CAPS) are driven by excitation voltages [3,4]. After demodulation, the angular position information is determined by a set of orthogonal sine and cosine signals. However, the output signals usually contain amplitude deviations, direct-current (DC) offsets, phase shifts, and other nonlinear disturbances which are difficult to handle.

To obtain more accurate angular position information, it is important to identify a solution to calibrate all the errors to the largest extent possible. This problem is generally formulated as an extraction of sinusoidal and cosine components from a signal in the simplified form of [2,3]:

(1) $$\begin{array}{l} y_{s}=a\cdot \sin\theta +b,\\ y_{c}=c\cdot \cos\left(\theta +\beta \right)+d. \end{array}$$

Based on the above expressions, many high-precision calibration methods have been proposed, while the application range generally does not include self-calibration scenarios. Le et al. [5] introduced a quadrature all-digital phase-locked loop method and proposed an interpolation technique to calibrate the angular position information. Nezar Abou Qamar et al. [6] presented the design of an auto-tuning output filter based on the interpolation method. In turn, Dhar et al. [7] proposed the establishment of an artificial neural network to compensate for angular errors. Tan et al. [8] used a radial-basis-function neural network for angle calibration.

To achieve the aim of self-calibration, many valuable methods have been developed. Hudson et al. [9] designed a new electronic nulling autocollimator to realize the calibration of the high-precision electronic nulling autocollimator. Lu [10] presented the time measurement Dynamic Reversal (TDR) method to realize self-calibration on the axis of angle encoders. Lu et al. [11] also introduced another self-calibration method for on-axis rotary encoders. The selection of a self-calibration algorithm constitutes another research direction. A self-calibration method based on the least-mean-square algorithm was first proposed in [12]. On this basis, the ellipse fitting method [13] and the optimization algorithm [14,15] based on gradient descent were developed. Improved iterative optimization algorithms were also developed to deal with problems in practical applications [16,17,18]. Additionally, Gao et al. [19] also proposed a new iterative algorithm for self-calibration. Additionally, Hou [20] introduced a self-calibration method based on a state observer, and Wu et al. [21] applied the technology to design a two-step gradient estimator.

The complete expression of signal model can be expressed as a Fourier series [18]:

(2) $$\begin{array}{l} y_{s}=\sum_{i=0}^{\infty }\left(a_{i}\cos\left(i\theta \right)+b_{i}\sin\left(i\theta \right)\right),\\ y_{c}=\sum_{i=0}^{\infty }\left(c_{i}\cos\left(i\theta \right)+d_{i}\sin\left(i\theta \right)\right). \end{array}$$

There are nonlinear error factors in the actual sensor signals, and the simple model that ignores the nonlinear part of the error makes the analysis and processing become achievable and simple. However, the analysis for simplified models is solely based on Equation (1), which is an approximate expression of Equation (2). The error factors that are ignored are not conducive to the further improvement of the accuracy of the sensor. If a suitable solution to deal with nonlinear factors can be found, signal extraction based on Equation (2) can yield higher calibration accuracies. In this study, a novel scheme is proposed to achieve the processing of nonlinear factors and can be used for the self-calibration of the complete nonlinear signal model.

The scheme is implemented based on the proposed model-based automatic search algorithm (MASA). The framework of the method is based and represented by signal flow networks (SFN). Linear layers were designed to represent the physical characteristics. In addition, an iteration approximation network (IAN) based on a fixed-point iteration method was proposed for the first time. It worked as a model search network and was embedded in the SFN. Regarding the search process, the least-mean-square-error was used to design the loss function of the network, and the Adam’s method [22] was applied as the optimizer.

For complex signal expressions that are difficult to handle, neural networks are used to re-model the signals, and the problem of solving signal parameters is transformed into a search problem for network parameters. Since the problem to be solved is a search problem in which the network input is unknown and the output can be known, numerical analysis techniques are used to fit a mapping relationship and convert the problem into a situation where the network input and output are known. Since the fitting scheme is unknown, an iterative approximation network is proposed, which is embedded in the calibration network so that the fitting scheme is also obtained by searching. This essentially proposes a solution to the problem of nonlinear equations in the case of unknown equation coefficients.

Compared with our previous work [19], the new research method is no longer limited to simple, definite model expressions but rather establishes a search process to achieve the fit of the model. A clear signal transfer function to complete the establishment and optimization of the signal network is needed in the previous work. For nonlinear signals with complex expressions in this study, the derivation of the transfer function is difficult or impossible to achieve. The previous work cannot effectively reduce the error. The newly proposed scheme establishes a search mechanism that can further reduce the error by completing the establishment of the transfer function and the construction of the signal network through the search method. The detailed results can be obtained from the simulation and experimental results that will be introduced next.

The remainder of this study is organized as follows: In Section 2, the error model of angular position sensors is discussed. In Section 3, the principle and specific implementation details of the proposed method are introduced. Section 4 then presents the simulation and experiments results, and a discussion of the results and our conclusions are outlined in Section 5.

## 2. Description of the Error Model

**Figure 1:**
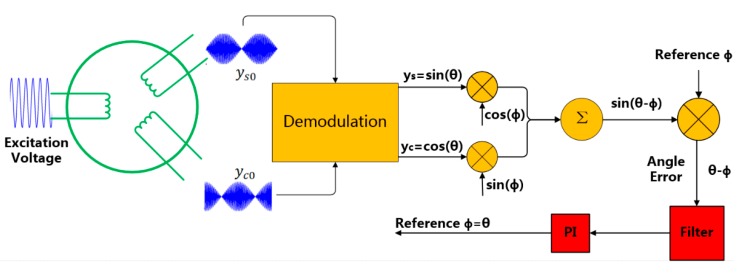
Working principle of angular position sensors.

Figure 1 shows the working principle of angular position sensors such as resolvers and capacitive angular position sensors. Under the influence of an excitation voltage, the sensors output two signals denoted as $y_{s0}$ and $y_{c0}$.

The gain coefficient of the sensor is defined as k, and $\omega_{e}$ and E are the frequency and the amplitude of the excitation voltage, respectively, while θ is the angle that needs to be measured. The two signals are modulated and can be expressed as [2,3]:

(3) $$\begin{array}{l} y_{s0}=k\cdot E\cdot \cos\left(\omega_{e}\cdot t\right)\cdot \sin\theta ,\\ y_{c0}=k\cdot E\cdot \cos\left(\omega_{e}\cdot t\right)\cdot \cos\theta . \end{array}$$

Ideally, the signals after demodulation [2,3] can be expressed as follows:

(4) $$y_{s}=\sin\theta ,y_{c}=\cos\theta .$$

In practical applications, the complete signal model that contains the interference factors is described according to Equation (2). Before further analyses of the model, mathematical formulas and theorems are used to simplify the problem.

$P_{n}(x)$ is defined as a polynomial with respect to x and the subscript n is the degree of the polynomial. By combining the binomial theorem [23] and de Moivre’s formula [24], cos(nθ) and sin(nθ) can be expressed as:

(5) $$\begin{array}{l} \cos\left(n\theta \right)=\sum_{i=0}^{\infty }\left(C_{n-i}^{i}+C_{n-1-i}^{i-1}\right){\left(-1\right)}^{i}2^{n-1-2i}\cos^{n-2i}\left(\theta \right),\\ \sin\left(n\theta \right)=\sum_{i=0}^{\infty }C_{n-1-i}^{i}{\left(-1\right)}^{i}2^{n-1-2i}\cos^{n-1-2i}\left(\theta \right)\sin\left(\theta \right). \end{array}$$

Equation (5) can be rewritten with the use of polynomial functions according to

(6) $$\cos\left(n\theta \right)=P_{n}\left(\cos\left(\theta \right)\right),\sin\left(n\theta \right)=\sin\theta \cdot P_{n-1}\left(\cos\left(\theta \right)\right).$$

Combining Equations (2) and (6), the expression of the two signals becomes

(7) $$\{y_{s},y_{c}\}=P_{\infty }\left(\cos\left(\theta \right)\right)+\sin\left(\theta \right)\cdot P_{\infty }\left(\cos\left(\theta \right)\right).$$

If the highest degree used is smaller than N, Equation (7) can be further expressed as:

(8) $$\begin{array}{l} y_{s}=\sum_{i=0}^{N}a_{i}\cos^{i}\left(\theta \right)+\sin\left(\theta \right)\cdot \sum_{i=0}^{N-1}b_{i}\cos^{i}\left(\theta \right),\\ y_{c}=\sum_{i=0}^{N}c_{i}\cos^{i}\left(\theta \right)+\sin\left(\theta \right)\cdot \sum_{i=0}^{N-1}d_{i}\cos^{i}\left(\theta \right). \end{array}$$

$y_{c}$ can also be expressed using the equivalent equation

(9) $$y_{c}=\sum_{i=0}^{N}c_{i}\cos^{i}\left(\varphi \right)+\sin\left(\theta \right)\cdot \sum_{i=0}^{N-1}d_{i}\cos^{i}\left(\varphi \right).$$

where φ=θ+β and β are the phase shifts of the two signals. Accordingly, the calibration method is introduced based on Equations (8) and (9).

## 3. Overall Description of MASA and Implementation Details

### 3.1. Signal Flow Networks and Self-Calibration Process

The transformation of nonlinear signals to standard orthogonal sine and cosine signals is the key concept for self-calibration and acquisition of angle information. This method designs a novel signal flow network to simulate the signal change process. The parameters of the transformation are represented by the nodes of the network. By establishing forward signal flow pipelines and backward optimization operation pipelines, the network can obtain accurate parameter values and extract the standard sine and cosine signals in the convergence state of the network.

**Figure 2:**
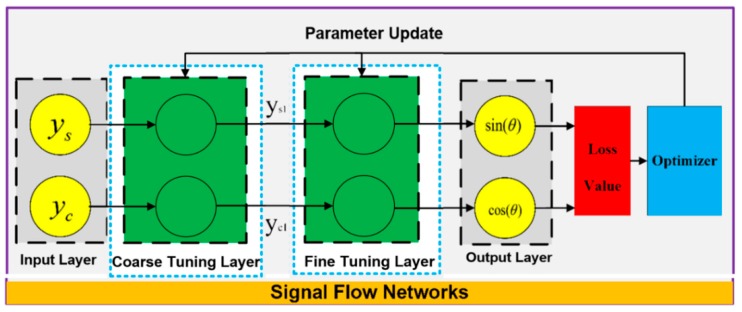
Schematic of the proposed method.

The proposed method mainly includes forward and backward operation pipelines. A schematic of the method is shown in Figure 2.

The forward operation pipelines start from the input layer. The values in the input layer are signals denoted by $y_{s}$ and $y_{c}$ in Equations (8) and (9). The coarse tuning layer deals with the coarse calibration of the input signal. The main linear errors such as the amplitude deviations and DC offset are suppressed. The output signals of the coarse tuning layer are denoted as $y_{s1}$ and $y_{c1}$. They can be expressed according to the following equations:

(10) $$\begin{array}{l} y_{s1}=\frac{y_{s}-a_{0}}{b_{0}}=\sin\left(\theta \right)+\sum_{i=1}^{N}a_{i}^{’}\cos^{i}\left(\theta \right)+\sin\left(\theta \right)\cdot \sum_{i=1}^{N-1}b_{i}^{’}\cos^{i}\left(\theta \right),\\ y_{c1}=\frac{y_{c}-c_{0}}{c_{1}}=\cos\left(\varphi \right)+\sum_{i=2}^{N}c_{i}^{’}\cos^{i}\left(\varphi \right)+\sin\left(\varphi \right)\cdot \sum_{i=0}^{N-1}d_{i}^{’}\cos^{i}\left(\varphi \right). \end{array}$$

The coarse calibrated signals are then input into the fine-tuning layer, which consists of the iteration approximation networks and the phase-shift-calibration layer. The output signals of the iteration approximation networks are defined as $y_{s2}$ and $y_{c2}$. In Section 3, it is proved that they can be expressed as:

(11) $$y_{s2}=\sin\left(\theta \right),y_{c2}=\cos\left(\varphi \right).$$

The phase-shift-calibration layer is another linear layer. The output signals are denoted as $y_{sf}$ and $y_{cf}$, and can be formulated as:

(12) $$\begin{array}{l} y_{sf}=y_{s2}=\sin\left(\theta \right),\\ y_{cf}=\frac{y_{c2}}{\cos\beta }+y_{c2}\cdot \tan\beta =\cos\left(\theta \right). \end{array}$$

The backward operation pipelines start from the loss value calculation. It deals with network optimization in the effort to ensure that the forward operation pipelines are accurate and reliable. The optimization target is referred to as a loss function. The value of the loss function is defined as:

(13) $$L={\left(1-{\left(y_{sf}^{2}+y_{cf}^{2}\right)}^{2}\right)}^{2}.$$

The optimizer is the engine of the backward operation pipelines, mainly containing the gradient calculation method and the parameter update rules.

The gradient calculation method is based on the back-propagation rule [25]. The parameter vector is defined as ψ, the input vector is defined as $Y_{i}={\left(y_{s},y_{c}\right)}^{T}$ and the output vectors of the coarse-tuning and fine-tuning layers are defined as $Y_{o1}={\left(y_{s1},y_{c1}\right)}^{T}$ and $Y_{o2}={\left(y_{s2},y_{c2}\right)}^{T}$. There exist mapping functions $f,\;f_{1},\;f_{2}$ and the following expressions:

(14) $$L=f\left(\psi ,Y_{o2}\right),Y_{o2}=f_{2}\left(\psi ,Y_{o1}\right),Y_{o1}=f_{1}\left(\psi ,Y_{i}\right).$$

According to the back-propagation rule [25], the gradient is calculated based on the formula:

(15) $$\frac{\partial L}{\partial \psi }=\frac{\partial f}{\partial Y_{o2}}\cdot \left(\frac{\partial Y_{o2}}{\partial \psi }+\frac{\partial Y_{o2}}{\partial Y_{o1}}\cdot \frac{\partial Y_{o1}}{\partial Y_{i}}\right).$$

The parameter update rules were designed based on the Adam’s optimization algorithm [22]. It has the ability to adjust the learning rate adaptively and has significant advantages over other random optimization algorithms [26]. The parameters are updated according to the following principles:

(16) $$\begin{array}{l} t\leftarrow t+1,m_{t}\leftarrow \beta_{1}\cdot m_{t-1}+\left(1-\beta_{1}\right)\cdot \frac{\partial L}{\partial \psi },v_{t}\leftarrow \beta_{2}\cdot v_{t-1}+\left(1-\beta_{2}\right)\cdot {\left(\frac{\partial L}{\partial \psi }\right)}^{2},\\ {\hat{m}}_{t}\leftarrow m_{t}/\left(1-\beta_{1}^{t}\right),{\hat{v}}_{t}\leftarrow v_{T}/\left(1-\beta_{2}^{t}\right),\psi_{t}\leftarrow \psi_{t-1}-\alpha \cdot {\hat{m}}_{t}/\left(\sqrt{{\hat{v}}_{t}}+\varepsilon \right). \end{array}$$

In Expression (16), the subscript t is the time step, $m_{t}$ is the updated biased first moment estimate, ${\hat{m}}_{t}$ is the bias-corrected first moment estimate, $v_{t}$ is the updated biased second moment estimate, and ${\hat{v}}_{t}$ is the bias-corrected second moment estimate. Parameter α is the step size, $\beta_{1}$ and $\beta_{2}$ are the exponential decay rates for moment estimation, and ε is a constant for numerical stability. The default settings are $\alpha =0.001,\beta_{1}=0.9,\beta_{2}=0.999,\varepsilon =10^{-8}$ [22].

During the process of continuous data collection, the backward optimization pipelines dynamically adjust the network parameters, and the forward operation pipelines obtain accurate calibration results.

### 3.2. Iteration Approximation Network and Convergence Analysis

The IAN was proposed to deal with signals in Equation (10). The mathematical basis of this network is a fixed-point iteration method [27]. The fixed-point iterative method is usually used for numerical fitting of nonlinear models [28,29].

**Figure 3:**
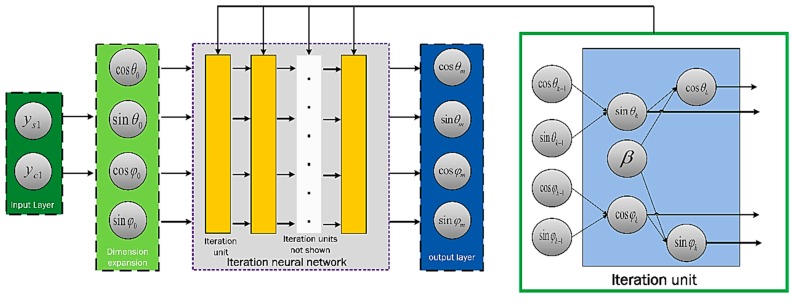
Schematic of the iteration approximation network.

The schematic of the iteration approximation network is shown in Figure 3. The initial value of the iteration process is equal to the output signal of the coarse calibration multiplied by a scaling factor such that the absolute value is less than unity. This is to prevent gradient divergence in the iterative process. Accordingly, $\sin(\theta_{k})$ and $\cos(\theta_{k})$ are defined based on the following iteration formulas:

(17) $$\begin{array}{l} \sin\left(\theta_{k}\right)=y_{s1}-\sum_{i=1}^{N}a_{i}^{’}\cos^{i}\left(\theta_{k-1}\right)-\sin\left(\theta_{k-1}\right)\cdot \sum_{i=1}^{N-1}b_{i}^{’}\cos^{i}\left(\theta_{k-1}\right),\\ \cos\left(\varphi_{k}\right)=y_{c1}-\sum_{i=2}^{N}c_{i}^{’}\cos^{i}\left(\varphi_{k-1}\right)-\sin\left(\varphi_{k-1}\right)\cdot \sum_{i=0}^{N-1}d_{i}^{’}\cos^{i}\left(\varphi_{k-1}\right),\\ \cos\left(\theta_{k}\right)=\left(\cos\varphi_{k-1}+\sin\theta_{k-1}\cdot \sin\beta \right)/\cos\beta ,\\ \sin\left(\varphi_{k}\right)=\sin\theta_{k-1}\cdot \cos\beta +\sin\beta \cdot \cos\theta_{k-1}. \end{array}$$

A parameter update behavior constitutes an iterative unit. The number of the iterative units in the fine-tuning layer is set to m.

The total nonlinear distortions are usually less than 1% [18]. Based on the assumption that the distortions of the parameters in Equation (17) are less than 0.01 and the expressions with small values are ignored, Equation (17) can be simplified to:

(18) $$\begin{array}{l} \sin\left(\theta \right)=g_{1}\left(\sin\left(\theta \right),\cos\left(\varphi \right)\right)=y_{s1}-\sum_{i=1}^{N}\left(a_{i}^{*}\sin^{i}\left(\theta \right)+b_{i}^{*}\cos^{i}\left(\varphi \right)\right)+ο\left(g_{1}^{m}\right),\\ \cos\left(\varphi \right)=g_{2}\left(\sin\left(\theta \right),\cos\left(\varphi \right)\right)=y_{c1}-\sum_{i=1}^{N}\left(c_{i}^{*}\sin^{i}\left(\theta \right)+d_{i}^{*}\cos^{i}\left(\varphi \right)\right)+ο\left(g_{2}^{m}\right). \end{array}$$

where $X={\left(\sin\left(\theta \right),\cos\left(\varphi \right)\right)}^{T},G\left(\sin\left(\theta \right),\cos\left(\varphi \right)\right)={\left(g_{1},g_{2}\right)}^{T}$. By considering the $L_{1}$ norm of $G\left(X_{1}\right)-G\left(X_{2}\right)$, the following inequality is established:

(19) $$\begin{array}{l} \left|g_{1}\left(X_{1}\right)-g_{1}\left(X_{2}\right)\right|=|\sum_{i=1}^{N}\left(a_{i}^{*}(\sin^{i}\left(\theta_{1}\right)-\sin^{i}\left(\theta_{2}\right)+b_{i}^{*}\left(\cos^{i}\left(\varphi_{1}\right)-\cos^{i}\left(\varphi_{2}\right)\right)\right)|\\ \leq \sum_{i=1}^{N}\left|a_{i}^{*}\right|\cdot \left|\sin^{i}\left(\theta_{1}\right)-\sin^{i}\left(\theta_{2}\right)\right|+\sum_{i=1}^{N}\left|b_{i}^{*}\right|\cdot |\cos^{i}\left(\varphi_{1}\right)-\cos^{i}\left(\varphi_{2}\right)|\\ \leq \sum_{i=1}^{N}\left|a_{i}^{*}\right|\cdot i\cdot \left|\sin\left(\theta_{1}\right)-\sin\left(\theta_{2}\right)\right|+|b_{i}^{*}\left|\cdot i\cdot \right|\cos\left(\varphi_{1}\right)-\cos\left(\varphi_{2}\right)|)\\ =L_{1}\cdot \left|\cos\left(\varphi_{1}\right)-\cos\left(\varphi_{2}\right)\right|+M_{1}\cdot \left|\sin\left(\theta_{1}\right)-\sin\left(\theta_{2}\right)\right|,\\ L_{1}=\sum_{i=1}^{N}|a_{i}^{*}|\cdot i\leq \sum_{i=1}^{N}0.01\cdot i=N\left(N+1\right)/200. \end{array}$$

With small phase shifts, $L_{1}\leq N\left(N+1\right)/200$ and $M_{1}\leq N\left(N+1\right)/200$.

The following expression can be obtained in the same way:

(20) $$\begin{array}{l} \left|g_{2}\left(X_{1}\right)-g_{2}\left(X_{2}\right)\right|\leq L_{2}\cdot \left|\cos\left(\varphi_{1}\right)-\cos\left(\varphi_{2}\right)\right|+M_{2}\cdot \left|\sin\left(\theta_{1}\right)-\sin\left(\theta_{2}\right)\right|,{\left|\left|G\left(X_{1}\right)-G\left(X_{2}\right)\right|\right|}_{1}\\ \leq \left(L_{1}+L_{2}\right)\cdot \left|\cos\left(\varphi_{1}\right)-\cos\left(\varphi_{2}\right)\right|+\left(M_{1}+M_{2}\right)\cdot \left|\sin\left(\theta_{1}\right)-\sin\left(\theta_{2}\right)\right|\\ \leq 0.01N\left(N+1\right)\left(\left|\cos\left(\varphi_{1}\right)-\cos\left(\varphi_{2}\right)\right|+\left|\sin\left(\theta_{1}\right)-\sin\left(\theta_{2}\right)\right|\right)\\ =L\cdot {\left|\left|X_{1}-X_{2}\right|\right|}_{1}. \end{array}$$

where L=0.01N(N+1) and L<1 when N≤9. The function $G\left(\sin\left(\theta \right),\cos\left(\varphi \right)\right)={\left(g_{1},g_{2}\right)}^{T}$ is a contraction map and the value of the function belongs to the range of the argument. According to the fix-point iteration theorem [28], the iterative equation has a fixed point, which is the solution of Equation (17). The iteration error satisfies the following expression:

(21) $${\left|\left|\varepsilon_{m}\right|\right|}_{1}\leq \frac{L^{m}}{1-L}{\left|\left|\varepsilon_{1}\right|\right|}_{1}.$$

where ε represents the iteration error, and the subscript represents the number of iterations. These derivations provide proof of convergence. In fact, the IAN can be considered to converge when the loss function converges in experiments.

## 4. Simulation and Experiment Results

### 4.1. Simulation for Feasibility Verifation

To verify the feasibility of the MASA method, a simulation experiment was conducted. The signal models were defined as:

(22) $$\begin{array}{l} y_{s}=1.10\cdot \sin\theta \cdot \left(1+0.02\cdot \sin\left(2\cdot \theta +1.00\right)\right)+0.01,\\ y_{c}=1.05\cdot \cos\theta \cdot \left(1+0.02\cdot \sin\left(2\cdot \theta +0.05\right)\right)+0.02. \end{array}$$

**Figure 4:**
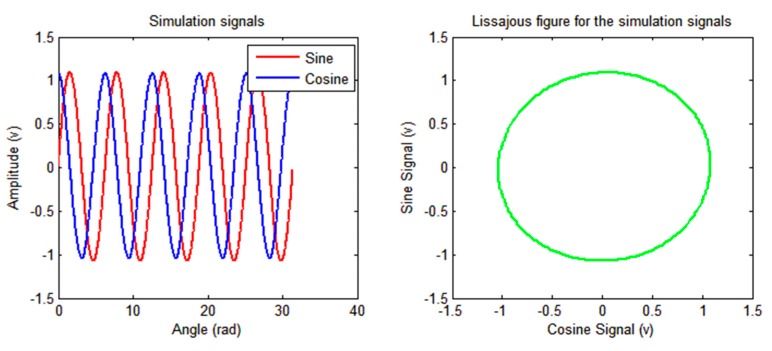
Time domain map and Lissajous figure of the signals.

**Figure 5:**
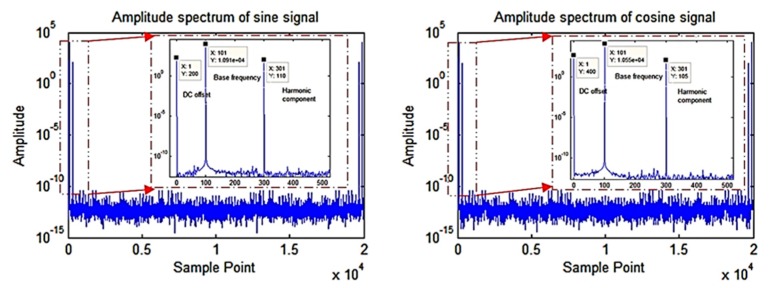
Amplitude spectrum of simulated sine and cosine signal.

The time domain map, the Lissajous figure, and the amplitude spectrums of the simulated signals are shown in Figure 4 and Figure 5.

The simulation data were randomly shuffled and then sent to the signal flow network to test the adaptability of the scheme in non-continuous sampling situations. The IAN deals with nonlinear harmonic components with orders smaller than three. The order of IAN was set to three, and the layer number was set to 14 to represent the fitting process.

In the network parameter initialization process, the maximum absolute values of the two output signals were used as the initial fundamental frequency coefficients, while the other parameters were initialized to zero. There are 20,000 sets of simulation data, and the parameter learning rate was set to 0.001. The other parameters of the optimizer were in accordance with the recommended values listed in [22]. Batch gradient descent was applied [30] and the batch size was set to 256. The optimization threshold was set to $1\times 10^{-14}$.

**Figure 6:**
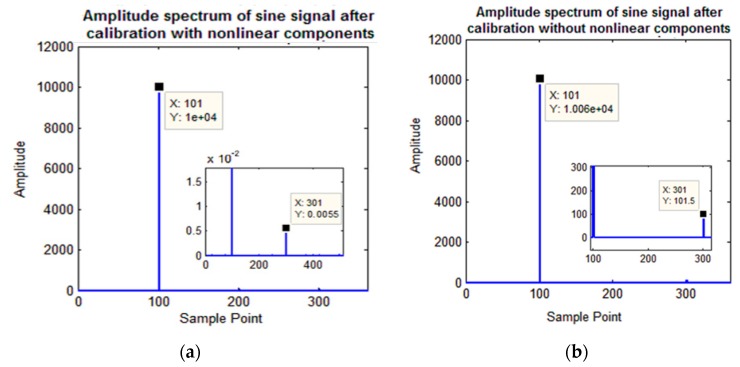
Amplitude spectrum of sine signals after calibration. (**a**) Proposed method; (**b**) method with simplified signal model.

Simulation experiments were based on the above parameter settings. The amplitude spectrum of the calibrated sine signal is shown in Figure 6a, and the amplitudes of other signal components are reduced to 0.005%.

In addition, self-calibration simulation experiments based on our previous work proposed in [19] with simplified signal model were also performed. The simulation results of two different schemes will be used to compare the superiority of the proposed scheme with respect to the previous scheme in terms of error suppression. The amplitude spectrum of the calibrated sine signal is shown in Figure 6b, and the amplitude of other signal components is reduced to 92.27%. The results show that the proposed scheme is better for the self-calibration of nonlinear signal models.

**Figure 7:**
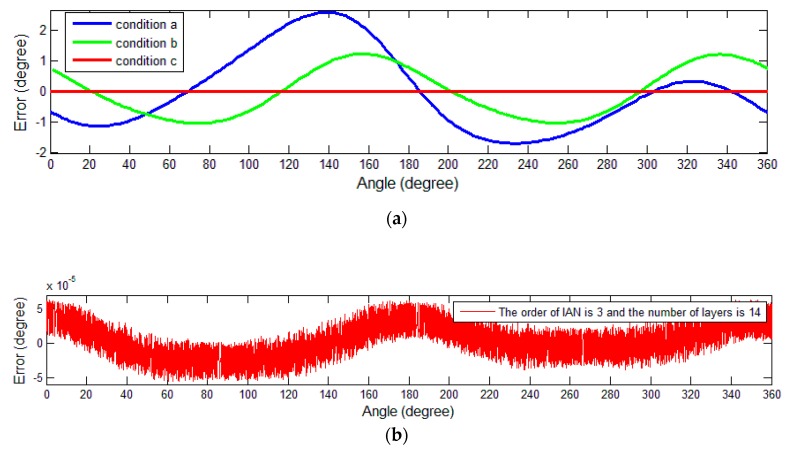
Angle errors in three tested conditions. (**a**) Display of the demodulation errors corresponding to the three tested conditions; (**b**) angle errors after calibration with the proposed method.

The residual values of the demodulation angle are depicted in Figure 7. The figure shows the angle error in three different conditions: Results without calibration (condition a), results after calibration using the method mentioned in [19] (condition b), and results after the calibration using the proposed method (condition c).

The results are also summarized in Table 1. The peak-to-peak value of the error and the maximum value of the absolute value are used as an error indicator of the angle. The error peak-to-peak value refers to the difference between the maximum value and the minimum value of the error, which is used to describe the variation range of the error; the maximum value of the absolute value describes the maximum level of the error. After the calibration using the method mentioned in [19], the peak-to-peak value of angle error was reduced to approximately 53%, whereas the value was reduced to approximately 0.0026% after the use of the proposed self-calibration process.

**Figure 8:**
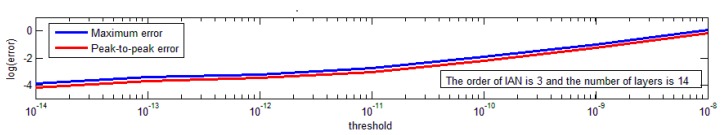
Relationship between threshold and angle error.

Ideally, as the optimization threshold decreases, the achievable angular accuracy increases. For further analyses, the relationship between the threshold and angle error is shown in Figure 8. The maximum error and the peak-to-peak error responses with respect to the threshold were fitted. The fitting formula is $\log\left(error\right)=p_{1}\cdot \log\left(threshold\right)+p_{2}$. The slopes for the maximum and peak-to-peak errors are −0.6450 and −0.6491, respectively.

The results indicate that under ideal conditions, a smaller optimization threshold will result in more accurate signals after the parameters converge. More analyses of other influencing factors are conducted in the experimental part.

### 4.2. Experimental Results

**Figure 9:**
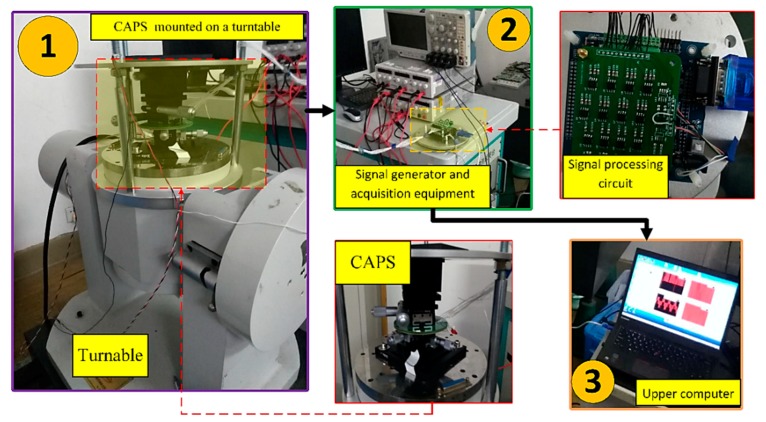
Experimental equipment, including the CAPS mounted on a turntable, signal generator, acquisition equipment, and a laptop computer.

For practical applications, experiments were conducted to verify the effectiveness and to study the factors that affect the accuracy of the proposed technique. The experimental equipment used for verification is illustrated in Figure 9.

In the experiment, the sensitive petal-form electrodes of the CAPS [3] are sine waves in polar coordinates spanning 36 cycles. The CAPS was mounted on a turntable which rotated at 0.5 °/s. The relationship between the angle information $\theta_{e}$ at each of the sensor output signals’ electrical cycle and the actual mechanical angle information $\theta_{m}$ is $\theta_{m}=\theta_{e}/36$. The frequency of $\theta_{e}$ is 0.05 Hz.

**Figure 10:**
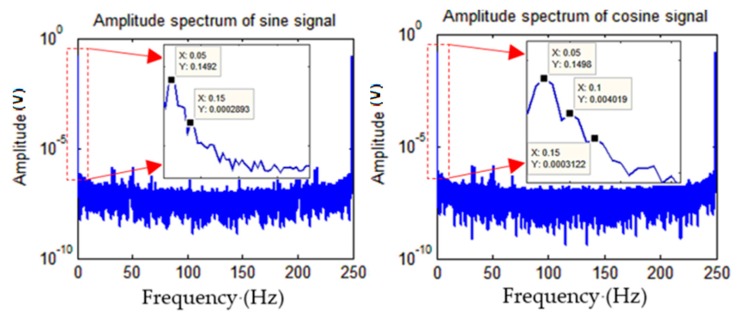
Amplitude spectrum of the collected sine and cosine data.

During the rotation process, the acquisition equipment processed the signals and sent the data to the laptop computer at a sampling frequency of 250 Hz. The acquisition time was 2 min, and a total of 180,000 data points were collected, which included 72 electrical angle periods and two mechanical angle periods. The exact values of the angle information were obtained through the turntable. Figure 10 shows the amplitude spectrum of the two sets of collected data.

Following calibration results were based on the 4th order IAN and the layer number was set to 14. In the network parameter initialization process, the maximum absolute values of the signals were used as the initial fundamental frequency coefficients, while other parameters were initialized to zero. The learning rate was set to 0.00001 and the other parameters were in accordance with the recommended values in [22]. The batch size was set to 256 and the optimization threshold was set to $1\times 10^{-8}$.

**Figure 11:**
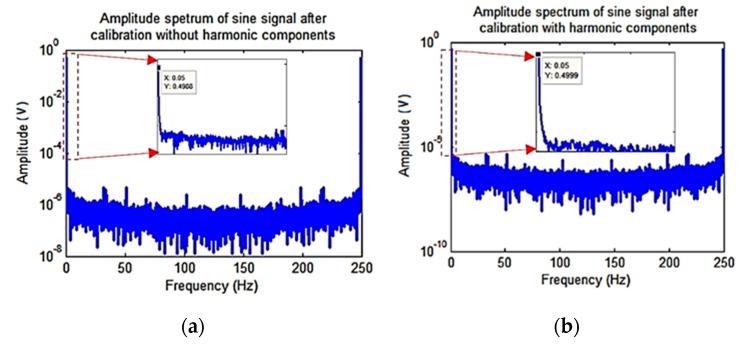
Amplitude spectrum of experimental sine signals after calibration. (**a**) Proposed method; (**b**) method with simplified signal model.

The amplitude spectrum of the calibrated sine signal after the use of the proposed method is shown in Figure 11a. The amplitude spectrum of the sine signal with the method mentioned in [19] is shown in Figure 11b.

**Figure 12:**
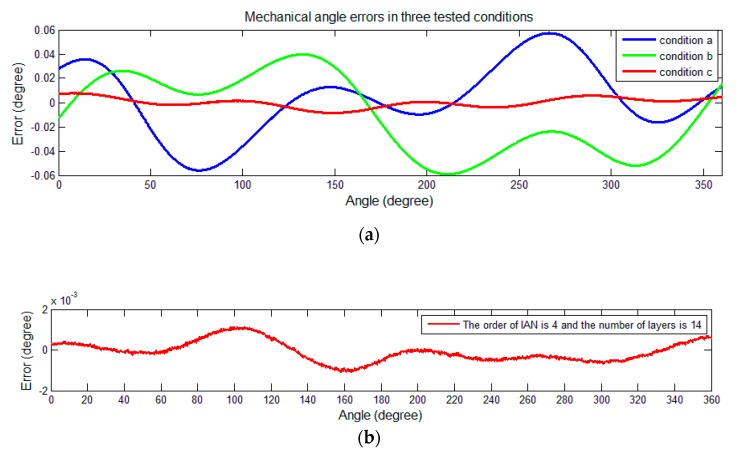
Mechanical angle errors in three tested conditions. (**a**) Display of the three demodulation errors; (**b**) mechanical angle errors using the proposed method.

According to the results shown in the spectrum, the amplitude component of the sinusoidal signal after calibration in the proposed scheme is 0.4999, which is close to 0.5 (ideal amplitude), while the value is 0.4988 after calibration with simplified model. For further analyses, the residual values of the mechanical angle are depicted in Figure 12. The three different tested conditions in simulation experiments are shown in Figure 12.

The detailed information is summarized in Table 2.

**Figure 13:**
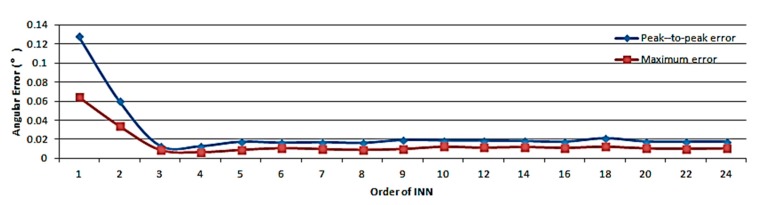
Variation of the angular error as a function of the order of IAN.

The value of the loss function cannot be reduced indefinitely in practical applications. When the optimal threshold is reached, the network structure becomes the main impact factor on the calibration results. The variation of the angular error as a function of the order of the IAN for a batch size of 256 and for 24 IAN layers with a learning rate of 0.00001 is shown in Figure 13.

The order of IAN has an influence on the nonlinear fitting accuracy. Higher orders may lead to improved accuracy, while they also result in a greater computational overhead and a slower convergence speed. According to the results of Figure 13, the accuracy of the output angle will be improved with the use of the 4th order IAN in association with the measured data.

**Figure 14:**
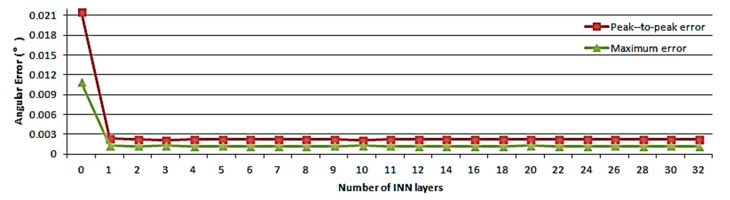
Variation of the angular error as a function of the order of IAN.

The number of IAN layers is another parameter used to evaluate the network fitting ability. It mainly reflects the iterative precision of the iterative formula. Figure 14 shows the relationship between the number of IAN layers and the calibration effect.

Ideally, more precise angular information can be obtained with additional layers. However, the network structure will become more complex and the convergence speed will be reduced. According to the results shown in Figure 14, the effect of the layer number on the improvement of the calibration accuracy is not as obvious as expected. On the basis of conforming to the accuracy requirements, the number of IAN layers, the parameter sizes, and the network complexity should be reduced to improve the convergence speed. The number of layers set in the experiment was 14 and corresponds to the case associated with the highest precision.

The results indicated that the proposed technology can reduce the peak-to-peak error and the maximum error to 1.76% and 1.63% respectively, while the conventional methodology that does not deal with nonlinear factors [19] can only reduce the errors to 93.80% and 86.02%. The basic building blocks of IAN determine the way in which nonlinear models are searched and fitted. In the experiment, sine and cosine functions were used as the basic units. The analysis of Figure 14 shows that the basic units may not be optimal. Different basic units result in different experimental results and there should be other more suitable units to achieve additional accuracy improvements.

## 5. Discussion for Computational Complexity

Time complexity and spatial complexity are often used to describe the computational complexity of an algorithm [31]. The spatial complexity is used to measure the size of the storage space temporarily occupied by the algorithm during operation, and the time complexity measures the running time of the algorithm.

For performance analysis, the amount of computation, which refers to the number of floating-point operations (without considering addition and subtraction operations) that occur when the model performs a forward propagation for a single input sample, is used to describe the time complexity. In the coarse adjustment layer, the input signal passes through the linear layer, and the number of floating-point operations is 2. In the fine-tuning layer, assuming that the IAN order is N and the number of layers is m, the number of floating-point operations required for each iteration unit can be calculated according to (17). The calculation results are as follows:

(23) $$\begin{array}{l} t_{1}=\sum_{i=1}^{N}(i+1)+\sum_{i=1}^{N-1}(i+1)+1=N(N+2),\\ t_{2}=\sum_{i=2}^{N}(i+1)+\sum_{i=0}^{N-1}(i+1)+1=N^{2}+3N-2,\\ t_{3}=3,\\ t_{4}=2.\\ t_{f}=t_{1}+t_{2}+t_{3}+t_{4}=2N^{2}+5N+3. \end{array}$$

For the model consisting of the coarse adjustment layer and an IAN with m layers, the total calculation amount defined as $t_{a}$ and the value is:

(24) $$\begin{array}{l} t_{a}=2+m\cdot t_{f}=(2N^{2}+5N+3)\cdot m+2,\\ t_{a}=O(N^{2}),\\ t_{a}=O(m). \end{array}$$

According to the results of Figure 13 and Figure 14, when the order and the number of layers are large, the calibration performance couldn’t be greatly improved, while the time complexity of the model is increased. This means that when the accuracy meets the requirements, it is necessary to reduce the order and number of layers as much as possible. In the experiment, N=4,m=14. The total calculation amount is about 772 floating point operations.

For spatial complexity, the total amount of memory access that occurs when the model completes a forward propagation process is used for analysis, which is expressed using the number of parameters of the model. The value of the total amount of memory access is 4N+3 according to Equations (8) and (9). In the experiment, the total amount of memory access is about 19 floating point numbers.

The order and number of layers of IAN affect the time complexity of the model. The order has a greater impact on time complexity and also affects the size of the parameters. The number of layers of IAN describes the number of iterations of fixed points. It does not affect the parameter size while mainly affects the calculation speed. The trade-off between calibration performance and computational complexity should be noted when the technique is applied.

## 6. Conclusions

Self-calibration of angle position sensors is commonly used and is necessary in practical applications to improve their accuracies when unknown input signals are used. This study proposed the use of the MASA method as a means of calibration. The self-calibration scheme was based on the nonlinear expression [2] and was achieved by combining the numerical analyses with iterative learning methods. The essence of this method is to reconstruct a signal expression model that can be searched to identify the parameters. This approach is more concerned with signal models than self-calibrating devices and is therefore likely to be applied to self-calibration of other devices.

Simulation experiments were also performed. The peak-to-peak and the maximum errors were reduced to 0.0026% and 0.0025% respectively, while the self-calibration for simplified model [19] reduced the errors to 53.00% and 47.42%, respectively. The relationship between the optimization threshold and the calibration accuracy was also analyzed, and the relationship curves and expressions under ideal conditions were obtained. Experiments were implemented in CAPS. The peak-to-peak and the maximum errors were reduced to 1.76% and 1.63% respectively, while the method for simplified model [19] reduced the errors to 93.80% and 86.02%. In addition, the order of IAN and the number of layers were also analyzed, and their effects on the calibration accuracy were determined. The optimal order and the optimal number of layers under experimental conditions were obtained. For the nonlinear problem discussed in this study, the key part of the method is to use the IAN architecture to characterize the nonlinear information. In the previous work [19], the nonlinear information required a clear functional expression to characterize. If the expression could not be found, only the simplified model could be processed and the nonlinear information had to be ignored.

For the proposed method in this article, the basic units and network structure in IAN need to undergo rigorous mathematical derivation. The construction method is complicated, while the experimental results showed that the advantages of the automatic search model for parameter adaptation cannot be fully utilized. Future research will focus on the automatic search of the signal expression architecture. To meet the real-time requirements, optimization of algorithmic computational complexity and hardware implementation will also be studied.

## Figures and Tables

**Table 1:** Angle errors in the three tested conditions based on simulations.

Category	Peak-to-Peak Error (°)	Proportion for Peak-to-Peak Error Reduction	Absolute Maximum Error (°)	Proportion for Absolute Maximum Error Reduction
Condition a	4.2980	/	2.6002	/
Condition b	2.2779	53.00%	1.2329	47.42%
Condition c	1.2711 × 10^−4^	0.0026%	6.4037 × 10^−5^	0.0025%

**Table 2:** Mechanical angle errors corresponding to the three tested conditions obtained from the experiments.

Category	Peak-to-Peak Error (°)	Proportion for Peak-to-Peak Error Reduction	Absolute Maximum Error (°)	Proportion for Absolute Maximum Error Reduction
Condition a	0.1274	/	0.0701	/
Condition b	0. 1195	93.80%	0.0603	86.02%
Condition c	2.24 × 10^−4^	1.76%	1.14 × 10^−3^	1.63%
